# Use of current automatic smoke evacuation system in flexible gastrointestinal endoscopy: Its feasibility and potential usefulness

**DOI:** 10.1002/deo2.367

**Published:** 2024-04-10

**Authors:** Yohei Nose, Motohiko Kato, Shoma Aoyagi, Kazunori Akeo, Kotaro Yamashita, Takuro Saito, Koji Tanaka, Kazuyoshi Yamamoto, Tomoki Makino, Tsuyoshi Takahashi, Yukinori Kurokawa, Hidetoshi Eguchi, Yuichiro Doki, Kiyokazu Nakajima

**Affiliations:** ^1^ Department of Next Generation Endoscopic Intervention (Project ENGINE) Graduate School of Medicine, Osaka University Osaka Japan; ^2^ Department of Gastroenterological Surgery Graduate School of Medicine Osaka University Osaka Japan; ^3^ Center for Diagnostic and Therapeutic Endoscopy Keio University School of Medicine Tokyo Japan; ^4^ AMCO Incorporated Tokyo Japan

**Keywords:** endoscopic submucosal dissection, flexible endoscopy, infection control, smoke evacuator, surgical smoke

## Abstract

**Objectives:**

Flexible endoscopy does not have a system that can automatically evacuate surgical smoke generated in the gastrointestinal lumen. We aimed to investigate the feasibility and potential usefulness of automatic smoke evacuation systems in flexible endoscopy.

**Methods:**

[Bench] After surgical smoke generated in the stomach was evacuated by the evacuator, the degree of residual smoke and gastric luminal collapse were evaluated to optimize the evacuator settings. [Animal] Insufflation, suction, and total operation time to complete the protocol of 10 cauterizations of the gastric mucosa were measured in three groups: “manual suction only,” “manual suction with automatic evacuation (50% force),” and “manual suction with automatic evacuation (70% force).” The stability of endoscopic visualization and operability was evaluated by 10 endoscopists blinded to those suction settings, and the number of manual suctions, insufflations, and total operation time were measured.

**Results:**

[Bench] The degree of residual smoke and gastric luminal collapse were inversely correlated. [Animal] When the automatic evacuator was partially used, there was no difference in the insufflation time, but the suction time (vs 50%; *p* = 0.011, vs. 70%; *p* = 0.011) and total operation time (vs. 50%; *p* = 0.012, vs. 70%; *p* = 0.036) were significantly reduced compared to manual operation only. Furthermore, manual suction with automatic evacuation (50% force) significantly improved the stability of endoscopic visualization and operability compared to manual operation only (*p* = 0.041, *p* = 0.0085).

**Conclusions:**

The automatic smoke evacuation in flexible gastrointestinal endoscopy was potentially feasible and useful by improving the device setting.

## INTRODUCTION

Vaporous or gaseous byproducts produced during electrocautery, laser surgery, or the use of ultrasonic scalpels are usually referred to as “surgical smoke”.^1^, ^2^ The surgical smoke generated by the energy‐based devices in flexible gastrointestinal (GI) endoscopic procedures, such as endoscopic submucosal dissection, obstructs the field of view and usually requires repeated manual suctions and insufflations by endoscopists.^3^, ^4^, ^5^, ^6^, ^7^, ^8^ In contrast, laparoscopic surgery already has systems that can automatically evacuate the smoke in the abdominal cavity,^9^, ^10^, ^11^ and it has already been reported, including ours, that the automatic smoke evacuator provides a better field‐of‐view and further reduces the risk of exposure to harmful compounds, including carcinogenic substances.^12^, ^13^, ^14^, ^15^ Other reports have shown that surgical smoke contains viruses such as human papillomavirus, and the use of smoke evacuators is recommended for laser surgery.^16^, ^17^ Furthermore, considering the risk of infection to healthcare workers in the era of coronavirus disease 2019 (COVID‐19),^18^, ^19^, ^20^, ^21^, ^22^, ^23^ a system that can evacuate surgical smoke even during endoscopic procedures may be important as a biohazard measure.^24^

While automatic smoke evacuators are already accepted as useful tools in the field of surgery, they are not currently used in the field of flexible GI endoscopy due to the following concerns. For one thing, they may cause GI tract luminal collapse, which may require continuous manual insufflation, and the physical properties of laparoscopy and GI endoscopy are different in nature, including insufflation method, target surgical field, volume, and pressure.^25^ Furthermore, there has been little discussion of surgical smoke evacuation in this field, even in terms of reducing the risk of surgical smoke. We understand the importance of fine‐tuning insufflation and suction, both of which are critical in GI endoscopy. However, if automatic smoke evacuation could be applied to the endoscopy, it might make the endoscopist's operation easier and further reduce the risk of exposure to surgical smoke. Although devices for laparoscopic surgery cannot be easily applied to GI endoscopy systems,^26^ the evacuator might be useful in the endoscopy if their settings are devised.

Therefore, this study aimed to investigate the feasibility and usefulness of a system that combines an automatic smoke evacuator for surgery and manual smoke evacuation under manual insufflation in a flexible GI endoscopic system.

## METHODS

First, the following benchtop experiments were conducted to optimize the settings of a commercially available automatic smoke evacuator for surgical use (IES3; ERBE Elektromedizin) in flexible GI endoscopy (in the stomach). This evacuator can be activated in conjunction with the output of the electrocautery when surgical smoke is generated. Smoke evacuation simultaneously output reduces the amount of smoke dispersal and obstruction of the field of view. The evacuator used in this study is the same in surgical procedures such as open and laparoscopic surgery. The suction force can be adjusted in the range of 10%–100% and is generally adjusted to 50%–70% in laparoscopic surgery, considering its possible impact on pneumoperitoneum. This evacuator consists of a pre‐filter (HEPA filter) and a 5‐layer main filter including a ULPA filter to reduce exposure to harmful substances to medical staff.^12^

### Bench testing

**FIGURE 1:**
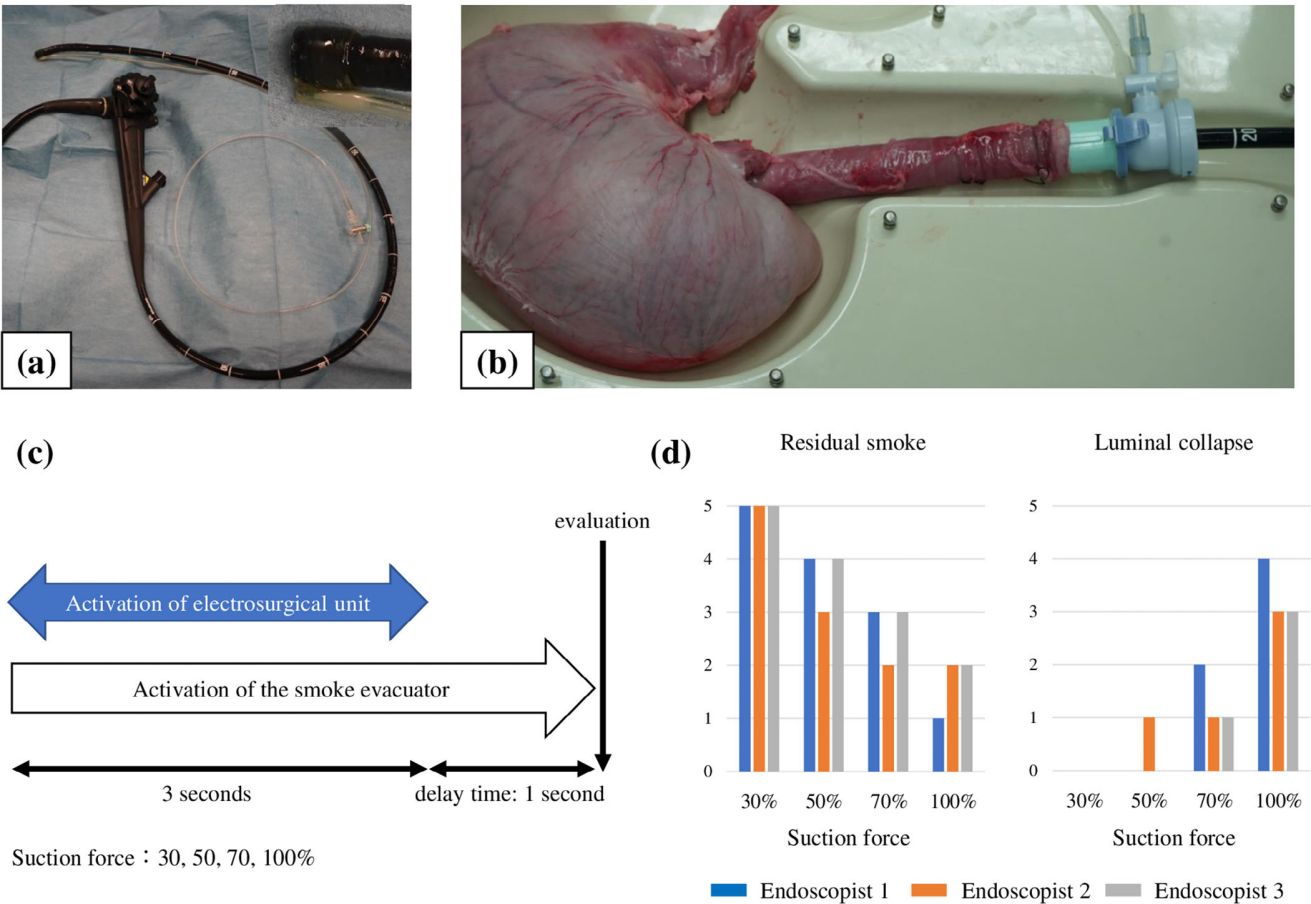
(a, b) The experimental setting in bench testing. A magnified view of the endoscope tip is included. (c) The experimental protocol. The suction force of the smoke evacuator was set to 30, 50, 70, or 100% with a delay time of 1 s. (d) The degree of residual smoke in the stomach and gastric luminal collapse after the end of the procedure. Three endoscopists blindly evaluated each item on a 6‐point scale from 0 to 5. Endoscopists 2 and 3 have more than 10 years of clinical experience, and 1 has less than 10 years.

The following experiments were performed on explanted swine stomachs. Since the evacuator cannot currently be connected directly to channels in the endoscope, an additional evacuation line (Impact Shooter, #16647; Top) was attached externally to the endoscope tip as an alternative, and the evacuator was connected to the evacuation line (Figure 1a,b). A flexible GI endoscope (GIF‐Q260J; Olympus Medical Systems) was then advanced into the stomach. A therapeutic electrosurgical probe (FlushKnife N‐S 2.5 mm, DK260J; FUJIFILM Medical Co., Ltd.) was connected to a high‐frequency electrosurgical unit (VIO300D; ERBE Elektromedizin). The mode of the unit was set to spray coagulation (Effect 2, Watts 30). The gastric mucosal surface was burnt with the probe for 3 s to generate surgical smoke in a reproducible fashion. The evacuator was activated at the same time as energization. In addition, a “delay time mode” was employed, which is a setting that allows smoke evacuation to continue at the same suction force for a set number of seconds after the end of energization (Figure 1c). During this time, an additional manual suction or insufflation was allowed ad‐lib. The degree of residual smoke in the stomach and gastric luminal collapse after the end of the procedure was evaluated on a 6‐point scale from 0 to 5 (Figure 1d). The suction force of the smoke evacuator was set to 30%, 50%, 70%, or 100% with a delay time of 1 s. Three endoscopists blindly evaluated these experiments.

Furthermore, the flow rate evacuated from the tip of the evacuation line attached to the endoscope was measured using an industrial flowmeter (#Thermal Mass Flowmeter 4043; Trans Tech Corporation). The length of the evacuation tube connecting the 1.3‐m evacuation line and the evacuator was adjusted as needed between 1 and 9 m, for a total length of 2.3–10.3 m. The conditions for tube thickness and stiffness were the same. Measurements were taken five times each with a flowmeter at 50% and 70% suction force of the evacuator (Figure S1).

### Animal studies

**FIGURE 2:**
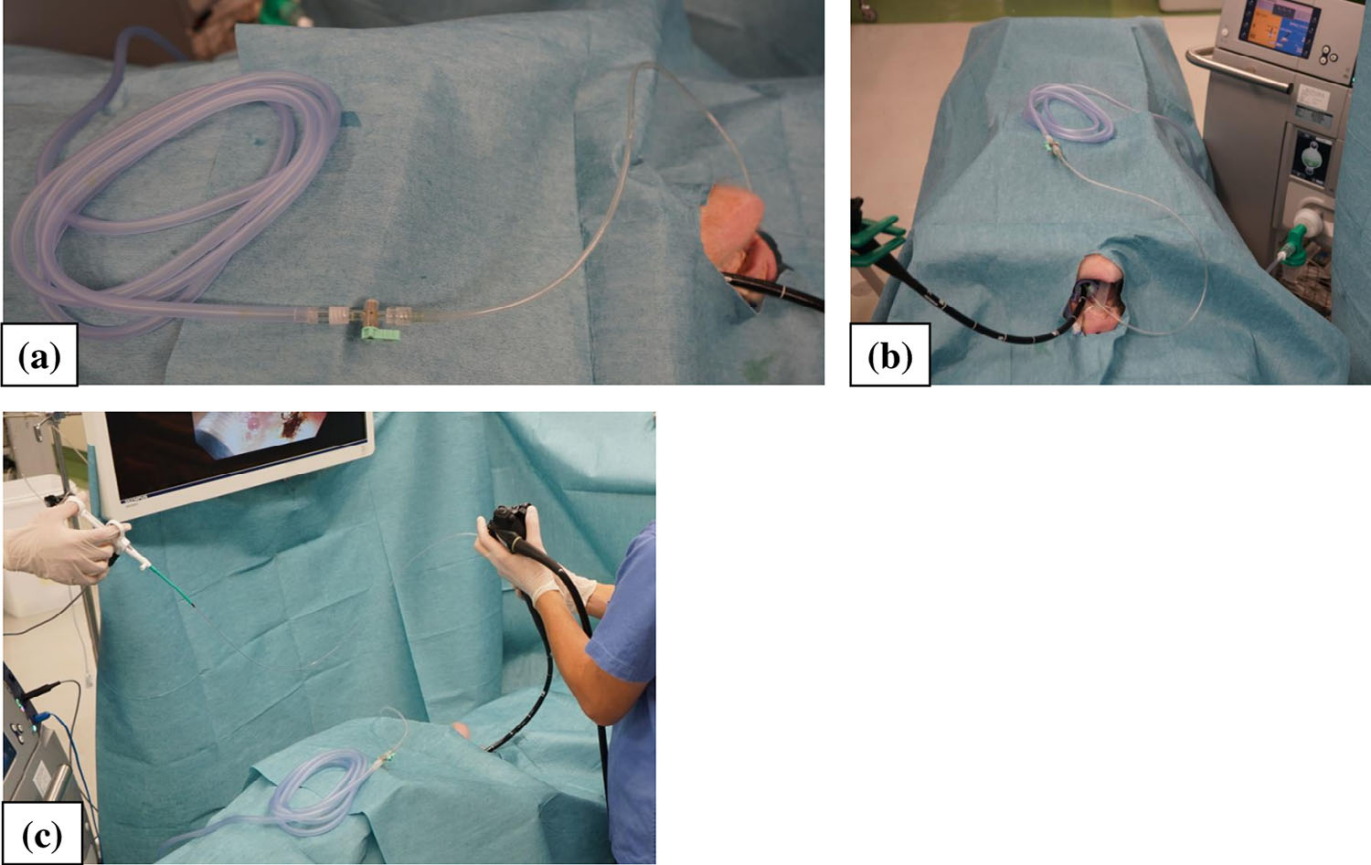
The experimental setting in animal study. A flexible gastrointestinal endoscope with an additional evacuation line was advanced into the stomach (a), and the evacuation line with a 3‐m evacuation tube was connected to the automatic evacuator (b). The actual operation is shown (c).

**FIGURE 3:**
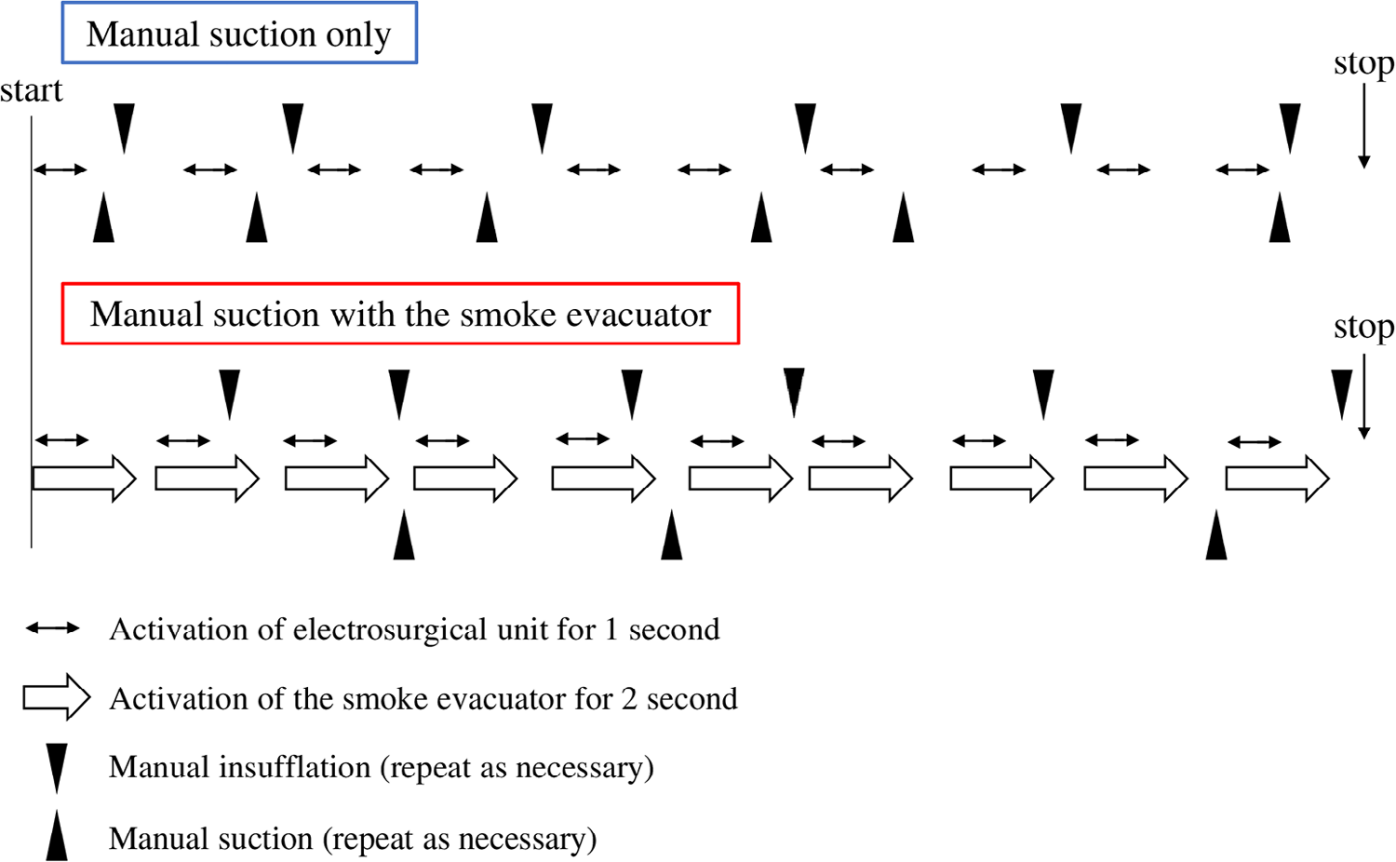
The experimental protocol. The gastric mucosal surface on the posterior wall of the antrum was burnt for 1 s to generate smoke. We defined one set as “10 repetitions of cauterization of gastric mucosa for 1 s.” During the operation, we manually repeated the suctions and insufflations as needed. These experiments were conducted five times in each of the three groups: manual suction only (only manual group), manual suction with 50% (50% semi‐automatic group), and 70% suction force of the smoke evacuator with a delay time of 1 s (70% semi‐automatic group).

[Experiment 1] All procedures were conducted using 3‐month‐old female pigs (average weight of 35 kg) under general anesthesia. Each pig was humanely euthanized upon completion of the experiment. Board‐certified surgeons conducted animal experiments with animals in the supine position. A flexible GI endoscope with an additional evacuation line was advanced into the stomach (Figure 2a). The evacuation line with a 3‐m evacuation tube was connected to the evacuator (Figure 2b). The gastric mucosal surface on the posterior wall of the antrum was burnt for 1 s to generate smoke in a reproducible fashion (Figures 2c and 3). Assuming an endoscopic submucosal dissection, we defined one set as “10 repetitions of cauterization of gastric mucosa for 1 s.”

Ten locations were randomly coagulated at least 5 mm apart. The first cauterization of the gastric mucosa was started when the endoscope was advanced into the stomach and the visualization was secured. One‐second cauterizations were repeated 10 times and suctions and insufflations were manually repeated based on each endoscopist's discretion until the total of 10 cauterizations were completed and the field of view was secured. The insufflation time, suction time, and total operation time per set were measured. The next cauterization was continued when each endoscopist judged that an optimal visualization was obtained and the procedure was feasible. These experiments were conducted five times in each of the three groups: manual suction only (only manual group), manual suction with 50% (50% semi‐automatic group), and 70% suction force of the evacuator with a delay time of 1 s (70% semi‐automatic group).

[Experiment 2] The same protocol as in Experiment 1 was used. Ten GI endoscopists were blinded to (1) only a manual group or (2) a 50% semi‐automatic group, and evaluated the stability of visual field and operability until completion of the protocol, as well as the number of manual suctions and insufflations and total operation time in each test. The stability of the visual field and operability were rated on a 6‐point scale from 0 to 5 immediately after the end of the procedure, with the highest score being 5 and the lowest score being 0. The animals and evacuator were covered with cloth, and a third party manipulated the evacuator, blindly performing this experiment.

### Statistical analysis

Differences between the groups were analyzed using the nonparametric Mann–Whitney U test. Paired T‐test was used when comparing data from the same endoscopists over time. All statistical analyses were performed using statistical software (JMP 14; SAS Institute Inc.). Statistical differences were calculated by using the Wilcoxon signed‐rank test. A *p*‐value < 0.05 was considered statistically significant.

## RESULTS

### Bench testing

The degree of residual smoke and gastric luminal collapse for four different levels of the suction force of the smoke evacuator, from 30% to 100% with a delay time of 1 s, was shown in Figure 1d. The residual smoke was objectively reduced with the smoke evacuator. The degree of residual smoke and gastric luminal collapse were inversely correlated. At 30% suction force, there was no significant luminal collapse, but there was a large amount of residual smoke. On the other hand, when the suction force was set to 100%, the residual amount of smoke was small, but the stomach collapsed easily. Therefore, the suction force of the smoke evacuator was 50%–70% with a delay time of 1 s was considered appropriate.

Furthermore, a graph of the flow rate from the tip of the evacuation line obtained using a flowmeter under conditions of 50% and 70% suction force of the smoke evacuator is shown in Figure S1. As we expected, the flow rate decreased as the tube was lengthened. Subsequent experiments were conducted with a 1.3‐m evacuation line connected to a 3‐m smoke evacuation tube. This was because a total length of 4.3 m was considered most suitable for the actual endoscopy unit arrangement in our daily practice.

### Animal studies

**FIGURE 4:**
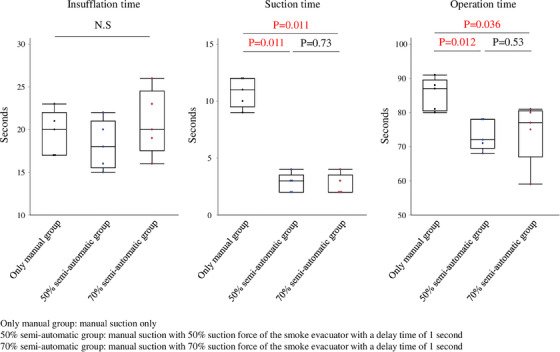
The insufflation time, suction time, and total operation time to complete one set for each operation measured in the following three groups: (1) manual suction only (only manual group), (2) manual suction with 50% suction force of the smoke evacuator with a delay time of 1 s (50% semi‐automatic group, (3) manual suction with 70% suction force of the smoke evacuator with a delay time of 1 s (70% semi‐automatic group). Each operation was repeated five times, and the significance of differences was calculated using the nonparametric Mann–Whitney U test.

[Experiment 1] When the smoke evacuator was partially used, there was no difference in the manual insufflation time (vs. 50% semi‐automatic group; *p* = 0.46, vs. 70% semi‐automatic group; *p* = 0.83), but the manual suction time (vs. 50% semi‐automatic group; *p* = 0.011, vs. 70% semi‐automatic group; *p* = 0.011) and total operation time (vs. 50% semi‐automatic group; *p* = 0.012, vs. 70% semi‐automatic group; *p* = 0.036) were significantly reduced compared with the only manual group. Comparing the 50% and 70% semi‐automatic groups, the 70% semi‐automatic group required longer manual insufflation time (*p* = 0.29) and total operation time (*p* = 0.53) than the 50% semi‐automatic group, but the difference was not significant (Figure 4). Based on these results, the next experiment was conducted with the suction force of the evacuator at 50%.

**FIGURE 5:**
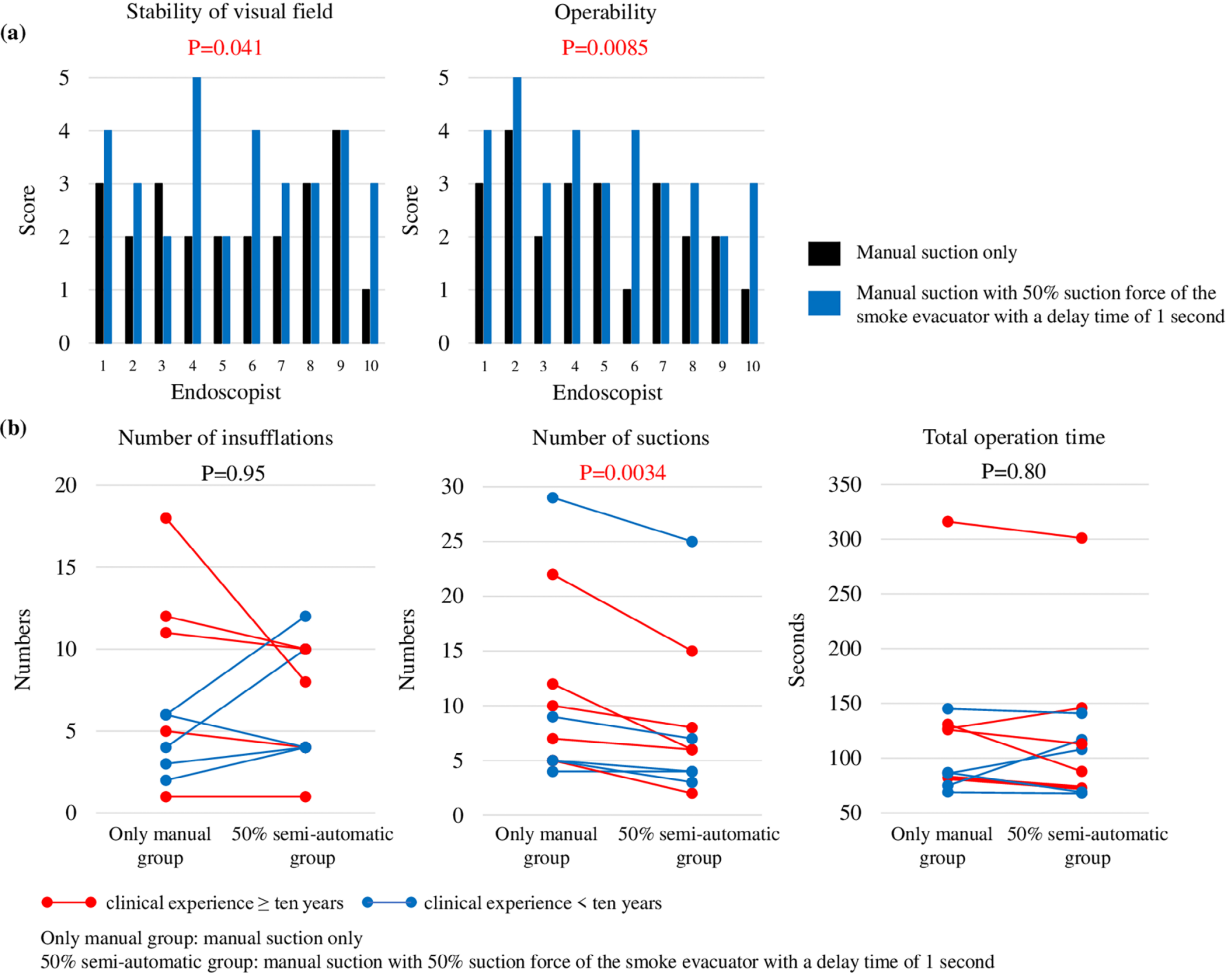
(a) The stability of visual field and operability. Ten gastrointestinal endoscopists were blinded to (1) manual suction only (only manual group) or (2) manual suction with 50% suction force of the smoke evacuator with a delay time of 1 s (50% semi‐automatic group). Each item was rated on a 6‐point scale from 0 to 5. Endoscopists 1, 4, 6, 7, and 10 have more than 10 years of clinical experience, and 2, 3, 5, 8, and 9 have less than 10 years. The significance of differences was calculated using the paired t‐test. (b) The number of insufflations, suctions, and total operation time for each operation method. Endoscopists with more than 10 years of clinical experience are shown in red, and endoscopists with less than 10 years are shown in blue. The significance of differences was calculated using the paired t‐test.

[Experiment 2] The stability of the visual field and operability were significantly improved in the 50% semi‐automatic group compared to the only manual group (*p* = 0.041, *p* = 0.0085, respectively, Figure 5a). The number of suctions was also significantly reduced when the smoke evacuator was used in combination (*p* = 0.0034), while the number of insufflations and total operation time showed no significant difference (*p* = 0.95, *p* = 0.80, respectively, Figure 5b). However, none of the endoscopists with more than 10 years of clinical experience increased the number of insufflations by using the evacuator (Figure S2). In animal experiments with the evacuator, no gastric luminal collapse occurred to the extent that the operation could not be continued if the settings of the evacuator were optimized. Therefore, no blind manipulation or adverse events were observed.

## DISCUSSION

The automatic smoke evacuator has not been available in the field of GI endoscopy mainly for the following three reasons. First, the evacuator may prevent endoscopists from performing the familiar manual insufflation and suction. Second, it may cause the GI tract lumen to collapse, resulting in a loss of field of view, and additional manual insufflation may be required, making the operation more complicated. Third, many endoscopists are unaware of the existence of the evacuator. Because of these concerns endoscopists still perform manual suctions and insufflations based on their judgment.^3^, ^4^ In contrast, laparoscopic surgery already has systems that automatically evacuate the surgical smoke,^9^, ^10^, ^11^ and surgeons do not need to perform suctions and insufflations themselves. In the future, the development of a system that can detect surgical smoke in the GI tract and automatically evacuate the smoke will lead to advances in endoscopic treatment. As a first step, we explored the feasibility and usefulness of a system that partially automates only the smoke evacuation.

We have developed a system that partially automates a GI endoscopy evacuation with the evacuator. We optimized the evacuation mode setting for the endoscopy and showed that the stability of the visual field and operability significantly increased and the actual number of manual suctions significantly decreased in combination with the evacuator. In a basic experiment using the flowmeter, the evacuator was able to evacuate approximately 35 ml of smoke‐containing gas per second locally from the end of the evacuation line at 50% suction force, which would have enabled effective smoke evacuation without gastric luminal collapse. In addition, the “delay time mode” of the evacuator allows evacuation to continue for a certain period after the end of energization, during which time manual insufflations can also be performed, and this may have led to improved operational simplicity. Thus, simultaneous insufflation and suction created a convection of smoke in the stomach, which may have improved the stability of the visual field and shortened the time required for the next cauterization. Although the number of seconds of delay time can be freely determined, all the delay times were set to 1 s in this study. There are two reasons for this. First, in the bench testing, the same experiment was conducted with the delay time set to 3 s, but luminal collapse was more likely to occur (data not shown). The second is that the setting of “10 repetitions of 1‐s cauterization” was used, and there was concern that setting the delay time to 2 s or longer would overlap with the next cauterization. Furthermore, using the evacuator significantly reduced the number of manual suctions. One reason may be that the combined use of the evacuator allowed the smoke to be evacuated at the same time as it was generated, which may have resulted in fewer major disruptions of vision compared to the manual repetition of suctions and insufflations. In contrast, there was no difference in the number of manual insufflations and total operation time. In fact, for four of the 10 endoscopists, the combination of the evacuator increased the number of manual insufflations, offsetting the effect of the decreased number of manual suctions, resulting in no difference in total operation time. The four endoscopists whose number of insufflations increased with the evacuator all had less than 10 years of clinical experience. Therefore, the individual's proficiency in endoscopic manipulation may have influenced the results. This is the first report showing that some amount of intraluminal surgical smoke can be effectively evacuated if the mode of the evacuator is properly adjusted. It should also be noted that the semi‐automatic evacuation system can be combined relatively easily with conventional manual suction and insufflation.

In surgery, the carcinogenicity of surgical smoke has long been considered a serious problem,^12^, ^14^, ^27^ and various smoke evacuators are commercially available.^12^, ^24^, ^28^, ^29^ In contrast, in the field of endoscopy, there is less awareness of the dangers of surgical smoke. Gas leakages from biopsy valves have been previously reported,^30^, ^31^, ^32^ and smoke evacuators with biological filters that can remove harmful substances may be useful to reduce the risk of exposure of medical staff to surgical smoke. In the current COVID‐19 era, awareness of smoke evacuation is expected to become increasingly important even in endoscopy. In the future, we hope that our study will lead to the development of systems that can automatically evacuate surgical smoke in flexible endoscopy and further advance GI endoscopic treatment.

We agree that this study has several limitations. First, this study was performed in a purely experimental setting, and because of the limited number of animals, we cannot exclude the possibility that our results are due to true biological variability; second, the number of endoscopists who participated was not so large. In the next study, we are planning to conduct a large blinded evaluation by the endoscopists with similar levels of experience; third, since there were no objective criteria to judge the stability of the visual field and operability with images, each endoscopist rated these items on a 6‐point scale based on their subjective criteria. There have been previous reports of the 6‐point scale in subjective evaluations.^33^, ^34^ Although this evaluation reduces the risk of using odd‐numbered steps, such as five, where a central answer is often chosen, it is important to establish a more objective evaluation method in the future. Additionally, the number of suctions and insufflations were counted, but the time required for each could not be evaluated because some endoscopists perform manual suctions and insufflations for extremely short periods; fourth, the experimental setting in this study does not necessarily reflect actual clinical endoscopic manipulation, and it is unclear whether it can be applied directly to clinical practice; fifth, there are differences in submucosal fatty issues between pigs and humans, and the generation of surgical smoke might be different.

In conclusion, the feasibility and potential usefulness of a system that partially automates smoke evacuation in flexible GI endoscopy were shown.

## CONFLICT OF INTEREST STATEMENT

None.

## ETHICS STATEMENT

This research is not medical research on human subjects and does not require IRB approval or written consent.

## Supporting information


**FIGURE S1** The flow rate evacuated from the tip of the evacuation line attached to the endoscope. Measurements were taken five times each repeatedly with a flowmeter at 50% and 70% suction force of the smoke evacuator (mean and 95% confidence interval).

**FIGURE S2** The number of insufflations, suctions, and total operation time for each operation method by endoscopist's clinical experience: manual suction only (manual) or manual suction with 50% suction force of the smoke evacuator with a delay time of 1 s (semi‐auto). Endoscopists with more than ten years of clinical experience are shown in red, and endoscopists with less than ten years are shown in blue. The significance of differences was calculated using the paired t‐test.
