# Supplementary figures and images for: Use of current automatic smoke evacuation system in flexible gastrointestinal endoscopy: Its feasibility and potential usefulness
Source: DEN Open. 2024 Apr 10;4(1):e367. doi: 10.1002/deo2.367 (PMC11007223; doi:10.1002/deo2.367)

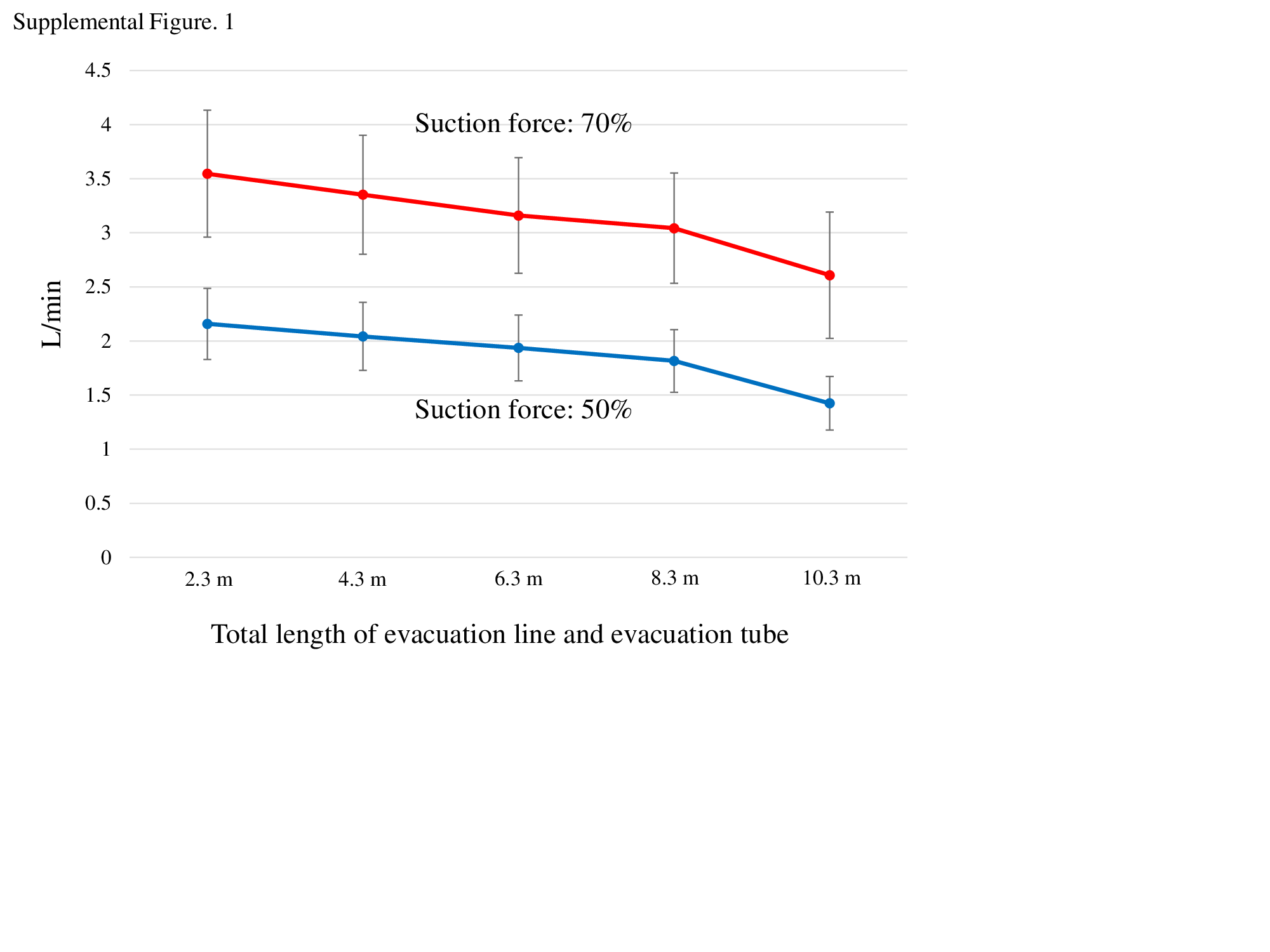

Supplement: Supplementary file 1 — FIGURE S1 The flow rate evacuated from the tip of the evacuation line attached to the endoscope. Measurements were taken five times each repeatedly with a flowmeter at 50% and 70% suction force of the smoke evacuator (mean and 95% confidence interval). [file DEO2-4-e367-s002.tiff]

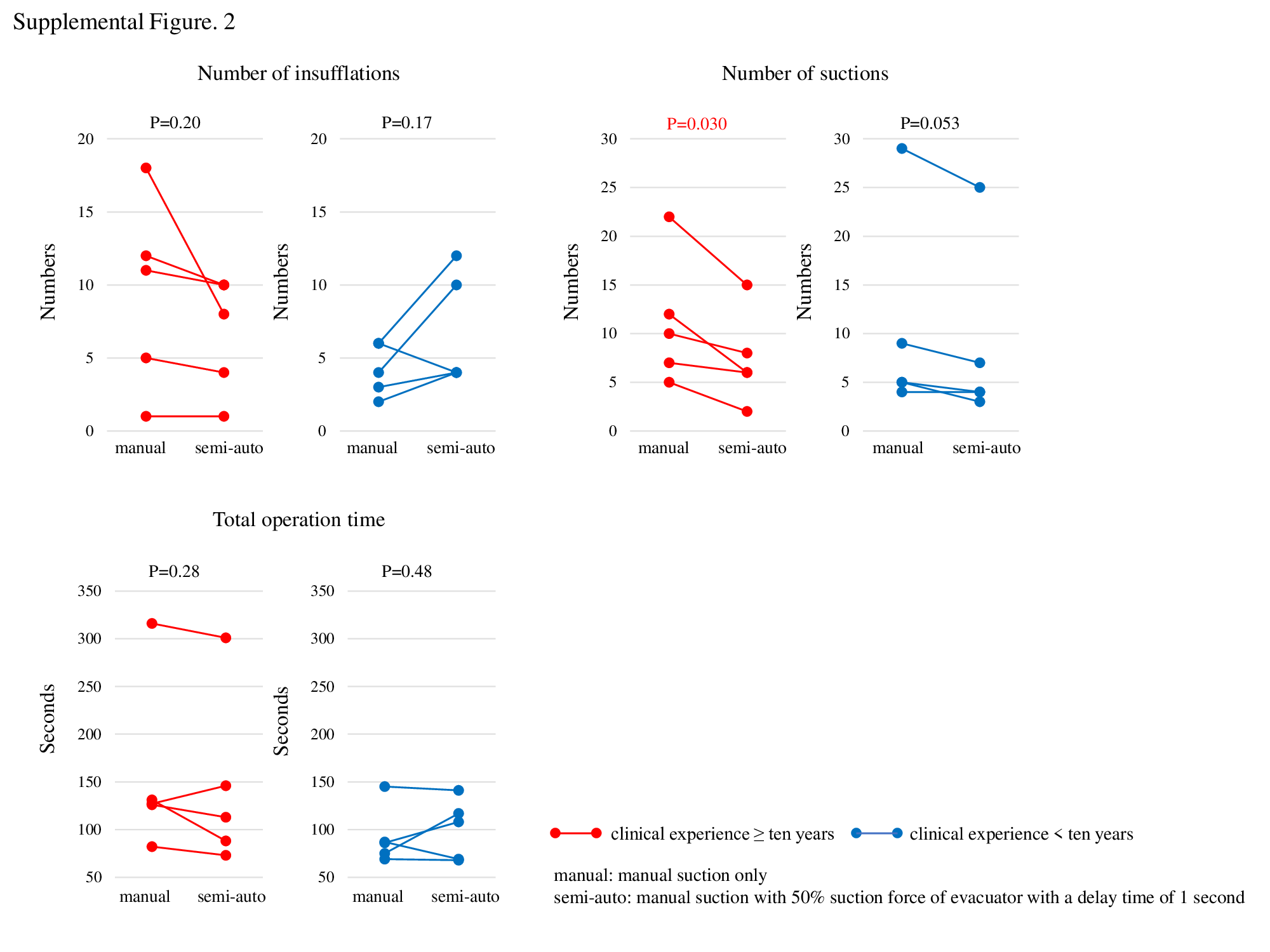

Supplement: Supplementary file 2 — FIGURE S2 The number of insufflations, suctions, and total operation time for each operation method by endoscopist's clinical experience: manual suction only (manual) or manual suction with 50% suction force of the smoke evacuator with a delay time of 1 s (semi‐auto). Endoscopists with more than ten years of clinical experience are shown in red, and endoscopists with less than ten years are shown in blue. The significance of differences was calculated using the paired t‐test. [file DEO2-4-e367-s001.tiff]
